# Bacterial Diversity in Old Hydrocarbon Polluted Sediments of Ecuadorian Amazon River Basins

**DOI:** 10.3390/toxics12020119

**Published:** 2024-01-31

**Authors:** Lara S. Corral-García, María Carmen Molina, Luis Fernando Bautista, Raquel Simarro, Carlos Iván Espinosa, Guillermo Gorines-Cordero, Natalia González-Benítez

**Affiliations:** 1 Centro de Investigación en Biodiversidad y Cambio Global, Department of Ecology, Universidad Autónoma de Madrid, C/Darwin, 2, 28049 Madrid, Spain; 2 Biodiversity and Conservation Unit, Department of Biology and Geology, Physics and Inorganic Chemistry, Instituto de Investigación en Cambio Global, Universidad Rey Juan Carlos, Tulipán s/n, Mostoles, 28933 Madrid, Spain; carmen.molina@urjc.es (M.C.M.); natalia.gonzalez@urjc.es (N.G.-B.); 3 Department of Chemical and Environmental Technology, ESCET, Universidad Rey Juan Carlos, Tulipán s/n, Mostoles, 28933 Madrid, Spain; fernando.bautista@urjc.es; 4 Plant Pathology Laboratory (DTEVL), INIA-CSIC, Ctra, de La Coruña, Km 7.5, 28040 Madrid, Spain; raquel.simarro@inia.csic.es; 5 Department of Biological and Agricultural Sciences, Universidad Técnica Particular de Loja, San Cayetano alto s/n, Loja 1101608, Ecuador; ciespinosa@utpl.edu.ec

**Keywords:** Ecuadorian Amazonia, river sediments, freshwater, hydrocarbon pollution, biomarkers, bacterial diversity

## Abstract

The Ecuadorian Amazon rainforest stands out as one of the world’s most biodiverse regions, yet faces significant threats due to oil extraction activities dating back to the 1970s in the northeastern provinces. This research investigates the environmental and societal consequences of prolonged petroleum exploitation and oil spills in Ecuador’s Amazon. Conducted in June 2015, the study involved a comprehensive analysis of freshwater sediment samples from 24 locations in the Rio Aguarico and Napo basins. Parameters such as water and air temperature, conductivity, soil pH, and hydrocarbon concentrations were examined. Total petroleum hydrocarbon (TPH) concentrations ranged from 9.4 to 847.4 mg kg^−1^, with polycyclic aromatic hydrocarbon (PAH) levels varying from 10.15 to 711.1 mg kg^−1^. The pristane/phytane ratio indicated historic hydrocarbon pollution in 8 of the 15 chemically analyzed sediments. Using non-culturable techniques (Illumina), bacterial analyses identified over 350 ASV, with prominent families including *Comamonadaceae*, *Chitinophagaceae*, *Anaeromyxobacteraceae*, *Sphingomonadaceae*, and *Xanthobacteraceae*. Bacterial diversity, assessed in eight samples, exhibited a positive correlation with PAH concentrations. The study provides insights into how microbial communities respond to varying levels of hydrocarbon pollution, shedding light on the enduring impact of oil exploitation in the Amazonian region. Its objective is to deepen our understanding of the environmental and human well-being in the affected area, underscoring the pressing need for remedial actions in the face of ongoing ecological challenges.

## 1. Introduction

International conservation programs, such as the Convention on Biological Diversity (CBD, 1992), the Nagoya Protocol (2010), and the more recent Kunming-Montreal Global Biodiversity Framework (2022), include among their goals the protection and conservation of biological diversity and a reduction in pollution risks to levels that do not harm biodiversity and ecosystem functions and services. This goal is a significant challenge for human societies, as it requires balancing the production of goods and services with the conservation of biodiversity, and is particularly important for countries with high biodiversity. One of the most significant non-renewable products for society is petroleum. However, petroleum exploration, extraction, processing, and transportation entail unavoidable pollution risks to the surrounding environment and human populations [1,2,3].

Among the different forms of current contamination, crude petroleum is considered highly hazardous for One Health (i.e., an integrated approach to balance the health status of people, animals, and the environment; WHO), since petroleum represents one of the most widespread pollutants with a variable toxic composition and persistence [4]. Once petroleum hydrocarbons have been released, the relative product composition may change due to both environmental conditions and physic–chemical transformation processes, as well as biotic degradation [5].

Simplifying the complex mixture of a crude oil spill, the lighter hydrocarbons will firstly be volatilized to the atmosphere in a short period of time, while the medium-weight compounds tend to be deposited in the soil or as a film on surface waters [6]. A third fraction of heavy hydrocarbons will be absorbed by the sediments lingering for many years at the site of release [7] and, with enough time, will suffer abiotic (mainly photo-oxidation, evaporation, and dissolution) and biotic degradation [8]. Napo basin crude oil is considered a heavy oil and, therefore, it is expected that for a long time after a spill, crude oil can still be present in an ecosystem [9]. The term total petroleum hydrocarbons (TPHs) is used to describe the mixture of chemicals that can commonly be found, including hexane, benzene, naphthalene, toluene, xylenes, fluorene, mineral oils, and gasoline components (ATSDR). TPHs quickly disperse into soil, groundwater, and surface water, hindering the passage of light and preventing habitual oxygenation, creating anoxic conditions, and causing an ecological impact on soil, benthic, and pelagic aquatic organisms [10]. Polycyclic aromatic hydrocarbons (PAHs) are a group of compounds that are composed of two or more fused benzene rings and are among the most widespread organic pollutants. PAHs are compounds of special concern because of their persistence and acute toxic, carcinogenic, and mutagenic potential [10]. PAHs accumulated in plant and animal tissues can find their way into the food web through bioaccumulation and biomagnification. Consequently, higher trophic organisms, including humans, can present several-orders-of-magnitude-higher PAH concentrations than the PAH concentrations measured in sediments or waters [11].

Microorganisms play an essential role in terrestrial biogeochemical cycles, agriculture, and human health [12]. However, local factors, such as temperature, solar radiation, exposure to water and wind, and the amount of petroleum, promote changes in hydrocarbon composition which, in turn, influence soil microbial activity and diversity [13,14]. Environmental hydrocarbons are metabolized differently by microorganisms, with n-alkanes being the first compounds biodegraded, then branched alkanes, and isoprenoids being the last ones [10,15]. Isoprenoids such as pristine (i-C_19_) and phytane (i-C_20_) are more resistant to microbial degradation than the alkanes n-C_17_ or n-C_18_ because they are weather-resistant. Thus, these hydrocarbons are a useful tool to estimate the grade of degradation and resistance to light and weathering [16,17]. The ratios pristine/n-C_17_, phytane/n-C_18_, and pristine/phytane are often used as petroleum biomarkers and can be used as a long-term indicator of anthropogenic pollution [10,17].

Recent advances in high-throughput technologies have enabled the development of various approaches for analyzing soil microbial community structures [18,19]. Nowadays, there is significant interest in studying the microorganisms present in contaminated environments for use in bioremediation. However, before adopting bioremediation, a comprehensive assessment of the crucial limiting parameters that affect efficacy is essential. This evaluation should take into account not only the physico-chemical properties of petroleum and environmental characteristics, but also the significant aspects of microbial community richness, diversity, and the interactions that govern the structure of the microbial community [7,20].

This research aims to investigate the impact of petroleum contamination on the bacterial community structure and biodiversity of the Ecuadorian Amazon, one of the most diverse areas in the world. Ecuador supports a wide variety of ecosystems and biomes, showing some of the greatest biodiversity and endemic species on the planet. The change in Ecuador from an agro-exporting country to an oil-exporting country with its economic system being based on this non-renewable resource has represented some of the most significant ecological, socio-economic, and cultural transformations in Ecuador’s history [21]. Despite the increasing awareness of the conservation and protection of biodiversity and its ecosystem services (BESs), Ecuador has been exploiting oil resources for more than 50 years. It is one of the countries most affected by the oil and mining industry in the world. Notwithstanding the fact that the Amazonian region is one of the priority areas for biodiversity conservation in the country, it is especially vulnerable to biodiversity losses [22], and it has been exploited by diverse petroleum companies such as Texaco and the Occidental Exploration and Production Company (OXY) from the 1970s up to now. From 1972 to 2015, more than 400,000 barrels of crude oil have been spilled into the Ecuadorian Amazon, with the Napo River basin being the area with the highest density of spills [23].

Most of the oil drilling exploitations in the Ecuadorian Amazon have polluted large areas of the territory, dumping highly toxic waste and wastewater into rivers and estuaries, in addition to the groundwaters and the atmosphere, affecting Ecuadorian Amazon BESs [24]. Part of the exploited Amazonian areas are located within important protected areas such as the Yasuní National Park and Biosphere Reserve, as well as the Limoncocha Biological Reserve, designated as Ramsar Wetland (UNESCO, 1998). The hydrologic systems of these protected areas are linked to the Aguarico, the Coca and the Napo River basins, and the Limoncocha Lagoon, along with other important Amazonian rivers such as Capucuy, Itaya, Jivino, and Indillana [25], which are inhabited by the Kichwa, Cofán, Waorani, and Ai’cofan indigenous populations [23,26]. Therefore, in addition to the ecological impact of petroleum pollution, there are socio-economic and health repercussions [2,27,28], as more than 40,000 inhabitants of the Napo, Coca, and Aguarico basins use rivers, lagoons, and estuarine waters as sources of drinking water, cooking, personal hygiene, agriculture irrigation, and fishing [29].

Given the vulnerability of these regions, the present study aims to conduct a preliminary analysis of the environmental health of the area. Therefore, the specific objectives are (i) to determine the composition and characteristics of petroleum hydrocarbons in river sediments and (ii) to describe the soil microbial communities exposed to different levels of hydrocarbon pollution.

## 2. Materials and Methods

### 2.1. Study Area

**Figure 1:**
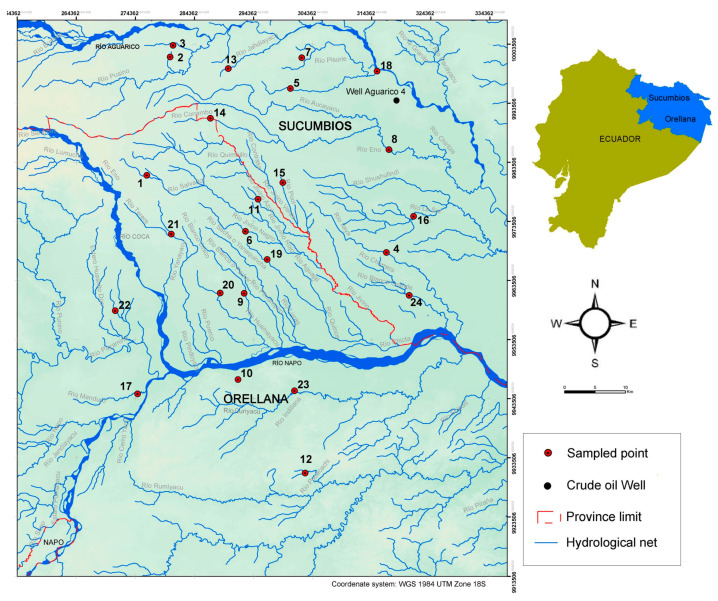
Study area with the sampled locations (red circles) and the crude oil well Aguarico 4 (black circle).

The study area was situated within the sub-basins of the Aguarico and Napo Rivers spanning the provinces of Orellana and Sucumbios, within the Ecuadorian Amazon rainforest. The selected area ranged in altitude from 200 to 550 m (m.a.s.l.) and was characterized by the Neotropical region covered by evergreen forest, featuring emergent trees exceeding 40 m in height and a canopy with an average height of 30 m. This region falls within a tropical rainforest climate, characterized by an average temperature of 25 °C and an annual rainfall exceeding 3200 mm [30]. The soil near the rivers is predominantly muddy and sandy and prone to seasonal flooding due to rainfall and fluctuations in the river’s water level (Figure 1).

### 2.2. Sample Collection

In June 2015, during the field expeditions, sediment samples were collected from 24 distinct points, each with three replicates (Figure 1, Table 1). The sampling procedure comprised three distinct phases. Initially, in the first phase, hydrocarbon concentrations (total petroleum hydrocarbons, TPHs, and polycyclic aromatic hydrocarbons, PAHs) were quantified across 24 different locations. Subsequently, during the second phase, bioindicator parameters (Pr/Ph, Pr/C_17_, and Ph/C_18_) were employed to ascertain the age of the oil contamination. These ratios serve as indicators since microbial and environmental degradation can impact them. Fourteen samples were analyzed in this phase and they were categorized into two groups based on TPH contamination levels: seven samples with low TPH concentrations (<90 mg kg^−1^) and seven samples with high TPH concentrations (>90 mg kg^−1^). In the final phase, the identification of microbial community structures in the different identified areas was addressed, covering the contamination risk spectrum evaluated by Espinosa et al. [2] in the same region, according to three risk levels: 3 samples with low pollution risk (0–0.3), 2 samples with medium risk (0.3–0.6), and 3 samples with high risk (>0.6) (Table 2). Through this comprehensive sampling design, we ensured the fulfillment of the study’s objectives by encompassing measurements of present hydrocarbon contamination (TPHs and PAHs), assessing microbial structures across the expected contamination risk spectrum, and determining the age of pollution. Sampling dredges were employed to collect sediment samples, each amounting to 100 cm³. Subsequently, all samples were air-dried for 72 h at room temperature, sieved (particle size > 2 mm), and stored in a light-free environment at 4 °C until shipment to Spain. Transportation of the samples was carried out using insulated containers with ice packs until they arrived at the laboratory; they were stored at −20 °C until they were analyzed.

### 2.3. Identification and Quantification of Total Petroleum Hydrocarbon and Polycyclic Aromatic Hydrocarbons

Total petroleum hydrocarbons (TPHs) were extracted from the 24 soil samples using a mixture of 1:1 vol/vol dichloromethane/acetone as the extraction solvent [31,32]. Briefly, 10 g of dry soil was placed in a 100 mL spherical flask and 25 mL of the extraction mixture was added. Then, the suspension was stirred for 30 min and the liquid was separated by decantation and the operation was repeated by adding 25 mL of fresh extraction mixture again. Both liquid phases were pooled and washed with water and the organic phase containing hydrocarbons was dried with anhydrous sodium sulphate and filtrated. Finally, the solvent mixture was allowed to evaporate under a gentle flow of nitrogen and TPHs were quantified by weight.

To extract polycyclic aromatic hydrocarbons (PAHs), 10 g of dried soil was stirred with 25 mL acetone for 15 min. After centrifugation, the liquid phase was separated, 25 mL of fresh acetone was added again to the soil, and the extraction was repeated. Then, 50 mL of *n*-hexane was added to the pooled extracts in a separation funnel and acetone and polar compounds were removed by adding and stirring with 100 mL water twice. The organic layer was dried with anhydrous sodium sulphate and filtrated. Quantification of PAHs was carried out by gravimetric measurement after solvent evaporation aided by a stream of nitrogen [31,33].

The hydrocarbon composition of extracted TPH samples was analyzed by gas chromatography–mass spectrometry (GC–MS) in a Bruker 450GC (Bruker Corp. Billerica, MA, USA) coupled to a Bruker 320 MS triple quadrupole mass spectrometer using electron ionization operating at 70 eV. Extracted TPH samples were diluted with carbon disulfide and filtered through a 0.45 µm nylon membrane. The GC was fitted with a Rxi-5Sil MS column (30 m length, 0.25 mm ID, 0.10 µm film thickness) (Restek. Lisses, France). The temperature in the column oven was programmed as follows: 80 °C for 2 min and then ramped to 320 °C at 10 °C/min, holding the final temperature for 5 min. The carrier gas was helium flowing at 1 mL/min. The injector temperature was maintained at 250 °C and 1 mL sample was injected in split mode (ratio 1:50). The temperature of the quadrupole was set at 150 °C and the temperatures of the ion source and the transfer line were 250 °C and 320 °C, respectively. Data acquisition and chromatogram processing were performed using the Bruker MS Workstation software v7.

Total petroleum hydrocarbons within the range of C_10_–C_40_ concentrations are presented in mg·kg^−1^. Ratios of *n*-heptadecane (C_17_) to pristane (Pr), *n*-octadecane (C_18_) to phytane (Ph), and pristane (Pr) to phytane (Ph) were determined for a total of 14 samples. These ratios were used as biomarkers of the microbial degradation degree [34] and age of petroleum spill [35]. The limit of detection of C_17_, C_18_, Pr, and Ph for the GC-MS analysis ranged from 0.005 to 0.02 µg/mL.

### 2.4. Microbial Biodiversity and Composition

To assess the microbial diversity and composition patterns, the Illumina MiSeq technique(Illumina Way, San Diego, CA, USA)was used in 8 samples (1,3,7,10,11,13,19,23) which represented the whole range of risk pollution [2]. DNA was extracted using the UltraClean Microbial DNA Isolation Kit (Mobio, Inc., Solana Beach, CA, USA). Purified DNAs were quantified by Picogreen and 0.5–3 ng of DNA was used for a first amplification of the 16SrRNA gene using primers (5′-ACACTGACGACATGGTTCTACACCTACGGGNGGCWGCAG-3′ and 5′- TACGGTAGCAGAGACTTGGTCTGACTACHVGGGTATCTAATCC-3′) which amplify the V3-V4 region of 16S. A second amplification was performed with primers (5′-AATGATACGGCGACCACCGAGATCTACACTGACGACATGGTTCTACA-3′ and 5′-CAAGCAGAAGACGGCATACGAGAT-[10 nucleotides barcode]-TACGGTAGCAGAGACTTGGTCT-3′) of the Access Array Barcode Library for Illumina Sequencers (Fluidigm). These primers contained a 5′ oligonucleotide tail used to allow sequencing in Illumina Mi-Seq PCR. Amplicons were denatured prior to being seeded on a flow cell, where clusters were formed and sequenced using a “MiSeq Reagent Kit v3” in a 2 × 301 pair-end sequencing run on a MiSeq sequencer. Pass filter reads were prepared using standard procedures (MiSeq Real-Time Analysis, Illumina). After being obtained, the fastq sequences were submitted to a quality control process using CutAdapt (v 3.5) [36] for sequencing adapter and primer removal, and FastP (v 0.23.2) [37] was used for quality trimming (Q20, minimal length of 50, trimming for both poly-x tails and paired-end adapters). FastQC (v 0.11.9) [38] and MultiQC (v 1.9) [39] were used for quality assessment both before and after trimming. Sequences were merged and clustered into ASVs (100% identity OTUS) and chimeras were detected and removed using the denoise paired function inside the dada2 [40] module in Qiime2 (v 2021.2) [41]. All forward and reverse sequences were truncated to position 250 to optimize merging. Taxonomy assignment was performed using the classify–consensus–blast method [42] in the feature classifier module in Qiime2, against the SILVA [43] release 138 99% similarity SSU database, considering only those assignments with a minimal consensus of 80%. The files containing this database’s sequences and taxonomy for this task were obtained from the Qiime2 v2021.2 resources’ official site.

## 3. Results

### Physico-Chemical Sediment Characterization

Water temperature exhibited a range of fluctuations, with a minimum recorded in the Pisuri River (sample 18) of 22.2 °C and a maximum of 34 °C in the Indillana River (sample 23) (Table 1). The mean water temperature across all samples was 24.9 °C. Notably, air temperature displayed a similar pattern of variation, with a maximum of 34 °C in the Eno River (sample 8) and a minimum of 22.7 °C in Anyiayacu (sample 3) (Table 1).

Conductivity, serving as an indicator of nutrient concentrations, exhibited significant variability across the study sites. The lowest recorded conductivity was 15.0 µS cm^−1^ in the Manduro River (sample 17), while the highest conductivity was observed in the Jivino Azul River (sample 11), measuring 138.5 µS cm^−1^ (Table 1). The average soil pH across all samples was 7.1, with pH values ranging from 6.3 in Manduro (sample 17) to 7.9 in the Pisuri River (sample 18) (Table 1).

The amount of TPHs and PAHs for each sample is summarized in Table 2. The highest TPH and PAH concentrations were measured in sample 9 in Blanco Chico River (847.4 mg kg ^−1^ and 711.1 mg kg^−1^, respectively). Sample 23, located in the Indillana River, also showed both high TPH and PAH concentrations (431.13 and 469.80 mg kg^−1^, respectively), and sample 10, which was located near the Napo River, showed a high TPH concentration (562.30 mg kg^−1^), but, although high, not so elevated PAH concentrations (134.07 mg kg^−1^) compared to samples 9 and 23. The lowest TPH concentrations were measured in sample 3 in the Anyiayacu River (9.40 mg kg^−1^) and the lowest PAH concentration was found in sample 4 in the Itaya River (10.15 mg kg^−1^) (Table 2).

**Figure 2:**
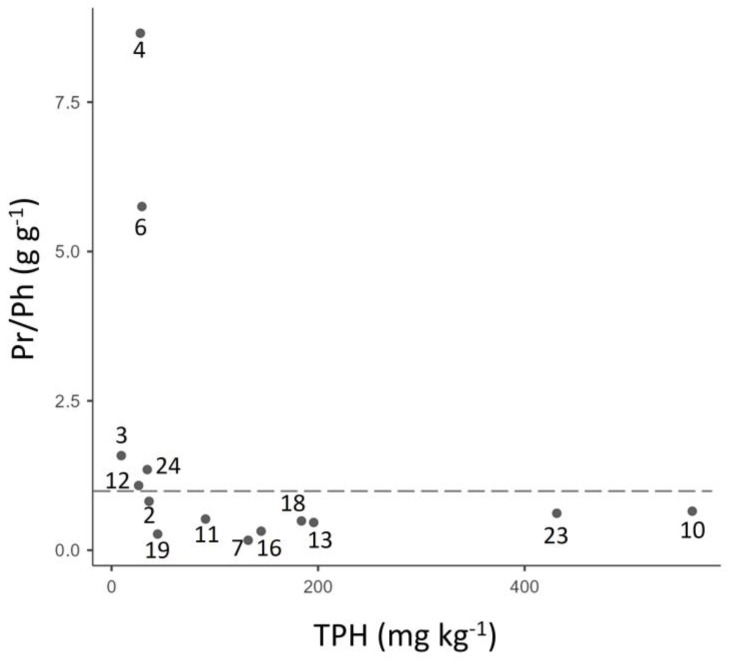
Pristane/phytane (Pr/Ph) ratio and the total petroleum hydrocarbon (TPH) concentrations (mg kg^−1^) at the different sampled points. The dashed line represents the fact that the Pr/Ph ratio is equal to 1.

The highest Pr/Ph ratio was found in the Itaya River (sample 4, 8.65) and the Jivino Negro River (sample 6, 5.75), whereas the lowest (0.17) was located in the Pisurie River (sample 7) (Table 2, Figure 2).

Higher Pr/Ph ratios corresponded to the lowest total petroleum hydrocarbon (TPH, mg kg^−1^) concentration samples (Figure 2). As shown in Table 2, the crude oil sample from Aguarico Well 4 has a Pr/Ph ratio close to 0. Most of our sampled sediments presented biomarkers of hydrocarbon pollution, such as pristane/phytane ratios (Pr/Ph) lower than or close to 1. Although 5 of the 14 soils analyzed had a Pr/Ph ratio higher than 1, only 2 of them, in the Itaya (sample 4) and Jivino Negro Rivers (sample 6), respectively, were higher than 3 (Table 2, Figure 2), and most samples showed Pr/C_17_ and Ph/C_18_ ratios lower than 1 (Table 2).

**Figure 3:**
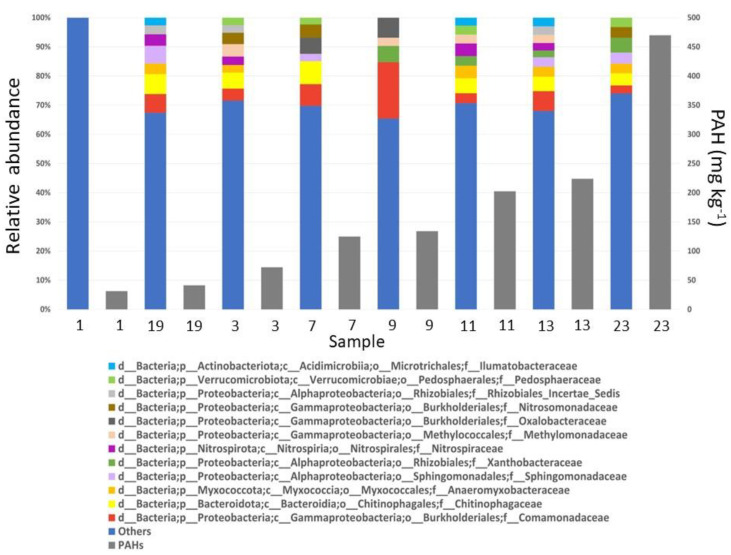
Microbial diversity (ASVs) at a family level (12 most abundant families) in each sediment sample (left axis) and the polycyclic aromatic hydrocarbon concentrations (PAH, mg·kg^−1^) in each sample (right axis).

Regarding bacterial diversity, more than 350 microbial ASVs from more than 40 phyla were identified using non-culturable techniques (Illumina) (Figure S1). The most abundant phyla were Proteobacteria, followed by Bacteroidota (Figure 3). The most abundant families across samples were mainly the following two: *Comamonadaceae* (Gammaproteobacteria class, Burkholderiales order) and *Chitinophagaceae* (Bacteroidia class, Chitinophagales order). Less abundant but also dominant were the families *Anaeromyxobacteraceae* (Myxococcia class, Myxococcales order), *Sphingomonadaceae* (Alphaproteobacteria class, Sphingomonadales order), *Xanthobacteraceae* (Alphaproteobacteria class, Rhizobiales order), *Nitrospiraceae* (Nitrospiria class, Nitrospirales order), and *Methylomonadaceae* (Gammaproteobacteria class, Methylococcales order) (Figure 3). Except for sample 1, the most abundant ASVs represent, on average, 40% of the total bacterial abundance, while the remaining 60% has been classified as “others”, with ASVs whose relative abundance is lower than 2%. 

**Figure 4:**
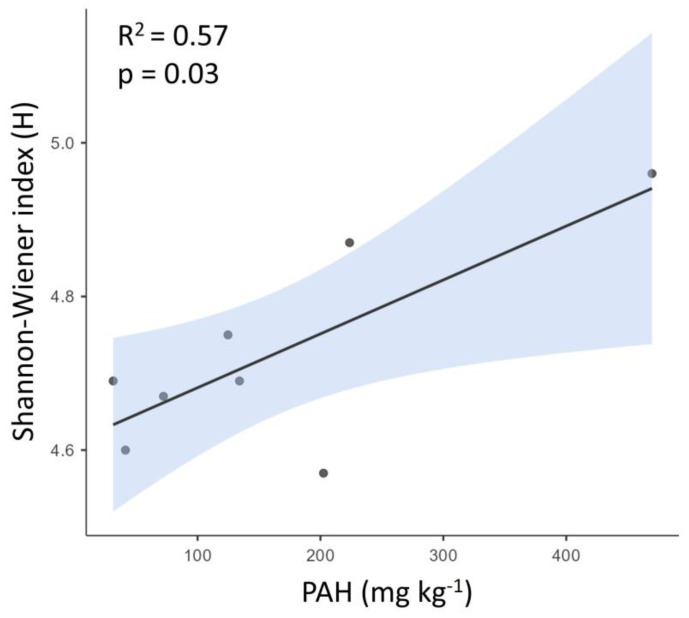
Linear relationship between the diversity Shannon–Wiener Index (H) and the polycyclic aromatic hydrocarbon (PAH, mg kg^−1^) concentrations in each sediment sample.

The bacterial diversity, calculated as the Shannon–Wiener Index (H) (Table 2), of those samples where the bacterial communities were addressed showed a significant linear and positive relationship with the PAH concentrations (Figure 4, R^2^ = 0.57, *p* < 0.05). The most polluted sample (sample 23, PAH = 469.80 mg·kg^−1^) showed the most diverse community (H) = 4.96 (Figure 4).

## 4. Discussion

In this study, both chemical and microbiological analyses were performed on sediments collected from different rivers within the sub-basins of the Aguarico and Napo Rivers in the Biological Reserve of Limoncocha of the Ecuadorian Amazon. Lower concentrations of TPHs were found to be correlated with higher Pr/Ph ratios. In fact, ratios higher than three suggest uncontaminated areas [44]. Therefore, samples 4 and 6 from the Itaya and Jivino Negro Rivers, respectively, were from non-polluted areas, whereas the remaining samples were exposed to a hydrocarbon source [45]. However, pristane/C_17_ and phytane/C_18_ ratios are biomarkers used to estimate the degree of microbial biodegradation and to assess the weathering state of the spilled hydrocarbon [10]. The pristane/C_17_ and phytane/C_18_ ratios are also good indicators of the extent of biodegradation, i.e., the age of the hydrocarbon [46]. The n-alkane compounds (C_17_ and C_18_, for example) are more easily decomposed by bacteria [45,47] than the isoprenoids pristane (C_19_) and phytane (C_20_), which are relatively more resistant to biodegradation due to their branched nature compared to the n-alkane linear forms [48]. Crude oil showed Pr/C_17_ (0.01) and Ph/C_18_ (0.18) ratios close to 0, while sediment samples showed that the mean Pr/C_17_ (0.57) and Ph/C_18_ (0.90) ratios were higher, but were still lower than 1, suggesting an old oil source, as isoprenoids were biodegraded a long time ago. These areas were intensely exploited in the early 1970s, making Ecuador a major oil exporter country in Latin America, with many extraction petroleum points [21]. Although Amazonian areas are still intensively subjected to hydrocarbon exploration, exploitation, transport, industrialization, and storage and commercialization activities, current exploitation oil wells are situated in other blocks, different from these study-sampled points.

Soil microbial diversity results indicated that Proteobacteria was the most dominant phyla (49%), followed by Bacteroidota (15.7%), Acidobacteriota (6.5%), Actinobacteriota (6.2%), and other minor groups such as Anaeromyxobacteraceae (Myxococcia) and Nitrospiraceae (Nitrospiria). The major groups detected in our sediments (except for Acidobacteriota) were also found in the bottom of the Kamenka River (Russian), which flows through oil and gas fields. These sediments were also highly contaminated with hydrocarbons, despite the absence of oil installations near the river [49]. Although less frequently, Acidobacteriota has also been associated with soils contaminated by petroleum hydrocarbons [50]. It is known that between 42 and 95% of bacteria of petroleum-contaminated soils belonged to phylum Proteobacteria, playing a crucial role in soils [17] and thus potentially being involved in petroleum hydrocarbon degradation. In the present research, *Comamonadaceae* (γ-Proteobacteria), *Sphingomonadaceae* (α-Proteobacteria), *Xanthobacteraceae* (α-Proteobacteria), and *Methylomonadaceae* (γ-Proteobacteria) were the dominant families within the Proteobacteria phylum. These results are consistent with previous studies in which these families were strongly represented in oil-contaminated soils [51,52,53,54,55,56,57,58,59]. Sydow et al. [60] showed how *Comamonadaceae* (β-Proteobacteria) increased in relative abundance by up to three orders of magnitude after exposure to branched alkanes. Members of this family have also been identified as degraders of aromatics [61,62]. An interesting diversification of this family has been found in environments contaminated with crude oil. This genetic diversification occurs concomitantly with an important functional diversification of catechol 2,3-dioxygenase genes, capable of degrading oil derivatives with limited oxygen environments such as oil fields [63], petroleum platforms [64], petroleum refinery waste sludge [65], or recirculated biofilter [66], although recently it has been shown that certain species in this group may be aerobic [67]. One of the most abundant families found in our samples was the *Sphingomonadaceae* (α-Proteobacteria), which has specific alkaline oxidation mechanisms for n-alkanes and is generally associated with a high capacity for polyaromatic hydrocarbon degradation [57,60,68]. Despite the *Sphingomonadaceae* family’s ability to degrade a wide range of aromatic compounds [69], recent genomic analyses have revealed dispersed biodegradation gene loci, posing challenges for genetic manipulation. This finding raises questions about their effectiveness in bioremediation, particularly for persistent aromatics in contaminated soils [70]. *Xanthobacteraceae* (α-Proteobacteria) from the Rhizobial order has been previously detected in hydrocarbon-contaminated locations [71], with a PAH degrading capacity [72]. Another family found in the most contaminated samples is the *Methylomonadaceae* (γ-Proteobacteria) formed by methylotrophs associated with environments that are methane-rich [73,74]. *Chitinophagaceae* (Bacteroidota) was found to possess the capacity to remove PAHs pyrene and benzo[a]pyrene [75] and metabolize oil [76]. Actinobacteria are oleophilic organisms frequently found in petroleum-contaminated soils (Nocardia genu) [77]. Nocardioides has a significantly higher degradation rate than other PAH-degrading bacteria and needs less time to break down these compounds [78]. *Anaeromyxobacteraceae* (Mixococcia) is an unusual myxobacterial genus that has been described in a wide variety of habitats due to its bioactive spectrum and secondary metabolites [79]. It has been identified in contaminated environments, playing a crucial role in the degradation and bioremediation of heavy-metal-contaminated sites [80,81]. *Nitrospiraceae* has been found in n-alkane-dependent thermophilic methanogenic enrichment cultures derived from production waters of a high-temperature petroleum reservoir [82]. Differences in the microbial compositions could be, at least partly, explained by the different PAH concentrations measured in the freshwater sediments. Although biodiversity has been reported to be negatively affected by hydrocarbon contamination [14,83], our results showed a contrary trend with PAH concentrations positively correlated with higher microbial diversity [84]. Interestingly, the less contaminated soil (sample 1) showed a completely different bacterial composition from the other more polluted samples, with no dominant or abundant families, but with many different families in very low relative abundance. Therefore, the stress caused in microbial soil populations by petroleum hydrocarbon pollutants usually results in natural selection. Those communities with the ability to survive under long periods of polluted conditions increased their abundance and therefore this led to an increase in the number of microorganisms with the potential to degrade petroleum hydrocarbons [85,86].

Sample 9 in the Blanco Chico River showed the highest concentrations of TPHs and PAHs. In fact, this sampling point is surrounded by potentially contaminating points such as oil and gas extraction blocks (PetrOriental, Auca Central, and Repsol), in addition to the influence of the Napo River. Sample 23, in the Indillana River, also showed high concentrations of TPHs and PAHs, while sample 10, at the south shore of the Napo River, showed higher concentrations of TPHs, but not as many PAHs as would be expected. In addition, these three samples were rated with high levels of contamination risk predicted by the model of Espinosa et al. [2]. This model is based on the accessibility of the contaminant, considering factors beyond the distance to the source of the spill, such as the area of contamination, the presence of runoff, and the flow of the watercourse, which influence the final impact of the contaminant. In these three areas (samples 9, 10, and 23), the high hydrocarbon concentrations may respond to the influence of the waters of the Napo River, which brings urban runoff from the second largest town in the Ecuadorian Amazon Region, San Francisco de Orellana [87]. However, lower PAH concentrations can also reflect a petrogenic origin derived from natural resources or a pyrolytic origin from hydrocarbon combustion [88,89]. It is known that complex mixtures of hydrocarbons, such as polycyclic aromatic hydrocarbons, have strongly hydrophobic characteristics, and therefore, PAHs tend to be deposited out of water and accumulate in the sediments [90]. However, petroleum compounds affect water quality, and therefore, this research wants to contribute to finding solutions to the problem generated by the presence of PAHs and TPHs in river sediments of the Ecuadorian Amazon basin. Nowadays, important advances in bioremediation to reduce water pollution are being developed. For example, the development of floating treatment wetlands (FTWs) significantly reduces water pollution as a consequence of the joint action of plant roots, endophytes, and biofilms associated with aquatic roots. This synergistic action is responsible for the adsorption, nitrification, denitrification, and degradation of organic compounds [91,92]. Adventurous and innovative chemical engineering techniques have been proposed, including the use of a novel Ca-Ag3PO4 capable of degrading phenanthrene with high efficiency [93]. These and other innovative techniques are promising, but a rigorous risk assessment is essential. In addition, factors such as target pollutant, initial load, operational strategies, and environmental conditions need to be considered. In river pollution where the river itself is the dispersing agent, processes such as FTW or chemical engineering techniques reduce their effectiveness, so the recommendation is for protection and conservation with enforceable legislation to ensure environmental and human health. Toxic oil spills and their derivatives directly affect microorganism composition and human populations exposed to this unhealthy environment [94]. To our knowledge, there are at least two Tilapias fish farms close to contaminated soils 7 and 16, respectively (Figure 1). According to the Yana Curi report [95], inhabitants of these areas are more likely predisposed to suffer from cancer, spontaneous abortions, and general deterioration of their health [2,95,96,97].

There has been a progressive increase in cancer cases among residents of the Orellana and Sucumbios provinces, with leukemia cases in children from 0 to 4 years old, three times more numerous in this area than in the rest of the country [94]. A significant proportion of men with anemia has also been detected in the sampled area [3]. Based on the aforementioned, it can be seen that activities related to the hydrocarbon industry have a series of difficulties that contribute, in one way or another, to weakening the fragile ecological and One Health balance of the Ecuadorian Amazon region.

## 5. Conclusions

Our research has unveiled a pervasive presence of hydrocarbon contamination in most of the freshwater sediments sampled in the Napo River basin, even in places where no contamination was expected to be detected a priori. This reflects the long-lasting impact of decades of oil exploitation in the Amazon region, probably amplified by the fluvial dynamics of these rivers. The correlation between bacterial biodiversity and PAH concentrations, with no similar trend observed for TPHs, highlights the specific sensitivity of microbial communities to certain pollutants. Furthermore, our study revealed substantial variations in microbial taxonomic diversity between unpolluted areas and those most severely impacted by oil pollution, underlining the profound ecological consequences of oil exploitation.

The Pr/Ph biomarker also indicated a long history of contamination in most of our samples, portraying a persistent environmental challenge that extends beyond the immediate time frame of our study. The problem has been compounded by recent spills such as the one in the Napo and Coca Rivers in the northeastern rainforest of Ecuador in 2020. This incident caused a major humanitarian and ecological catastrophe, adversely affecting indigenous communities inhabiting the region. These communities, being the most impacted by the environmental crisis, continue to demand a prompt and comprehensive response to mitigate the damage caused by oil exploitation.

## Figures and Tables

**Table 1:** Geographic coordinates and physical and chemical parameters of sampled locations: air and water temperature (°C), dissolved oxygen (mg L^−1^), soil pH, and conductivity (µS cm^−1^). * Samples where microbial composition was analyzed using the Illumina MiSeq technique.

Sample ID	River or Location	Latitude (N)	Longitude (E)	Air T (°C)	Water T (°C)	OD (mg L^−1^)	Soil pH	Conductivity (µS cm^−1^)
1 *	Locatayacu							
2	Locatayacu	0.0181	−76.9700	22.8	23.2	7.5	7.3	64.2
3 *	Anyiayacu	0.0299	−76.9711	22.7	23.2	7.4	7.3	57.7
4	Itaya	−0.2903	−76.6401	33.1	26.7	6.2	7.2	133.7
5	Aguas Blancas	−0.0364	−76.7920	-	23.6	6.6	7.1	36.3
6	Jivino Negro	−0.2558	−76.8599	31.5	24.8	6.2	7.4	111.2
7 *	Pisurie	0.0107	−76.7743	25.2	23.5	5.4	6.7	17.7
8	Eno	−0.1224	−76.6497	34.0	25.3	6.5	7.1	102.5
9	Blanco Chico	−0.3500	−76.8641	28.6	24.8	7.1	7.4	88.7
10 *	Napo	−0.4805	−76.8725	27.9	24.2	5.4	7.0	37.7
11 *	Jivino Azul	−0.2058	−76.8409	30.8	24.7	6.6	7.4	138.5
12	Pucacachi	−0.6013	−76.7784	28.4	24.3	6.6	6.6	15.9
13 *	Jandiyacu	−0.0059	−76.8859	27.0	23.9	7.4	7.3	48.5
14	Conambo	−0.0820	−76.9133		24.6	6.3	7.2	96.9
15	Harbent	−0.1804	−76.8035	29.7	24.4	7.5	7.7	127.4
16	La Sur	−0.2416	−76.6133	28.7	25.3	6.5	7.0	127.9
17	Manduro	−0.5023	−77.0263	26.3	23.6	5.5	6.3	15.0
18	Pisuri	−0.0098	−76.6602	25.7	22.2	7.9	7.9	66.8
19 *	Sacha	−0.2982	−76.8271	33.3	25.2	6.0	7.1	92.2
20	Huamayacu	−0.3496	−76.8983	27.6	25.7	6.9	7.6	122.2
21	Blanco	−0.2593	−76.9728	28.7	24.8	4.4	6.9	49.5
22	Huachito	−0.3765	−77.0578	25.9	24.5	6.4	7.4	88.9
23 *	Indillana	−0.4916	−76.7855	26.1	34.0	5.0	6.4	18.0
24	Blanco Grande	−0.3530	−76.6113	27.9	25.3	4.9	7.2	106.2
Well Aguarico 4	Tena province	−0.0633	−76.6375					

**Table 2:** Total petroleum hydrocarbons (TPHs, mg·kg^−1^), polycyclic aromatic hydrocarbons (PAHs, mg·kg^−1^), pristane/phytane ratio (g·g^−1^), pristane/C_17_ (g·g^−1^) and phytane/C_18_ (g·g^−1^), bacterial richness (Z), Shannon diversity indexes, and risk pollution values. * Samples where microbial diversity and composition were analyzed using the Illumina MiSeq technique.** Risk model variables: friction surface, accessibility to petroleum waste, and contamination potential [2].

Sample ID	River or Location	TPH (mg·kg^−1^)	PAH (mg·kg^−1^)	Pr/Ph (g·g^−1^)	Pr/C_17_ (g·g^−1^)	Ph/C_18_ (g·g^−1^)	Richness (Z)	Shannon (H)	Pollution Risk **
1 *	Locatayacu	70.57	31.30				247	4.69	0.00
2	Locatayacu	36.30	290.90	0.82	0.31	0.70			0.00
3 *	Anyiayacu	9.40	72.20	1.58	0.39	0.42	237	4.67	0.00
4	Itaya	27.90	10.15	8.65	1.77	0.59			0.38
5	Aguas Blancas	188.80	52.53						0.67
6	Jivino Negro	29.40	21.50	5.75	0.52	0.10			0.82
7 *	Pisurie	132.30	124.70	0.17	0.28	1.36	233	4.75	0.61
8	Eno	20.40	25.70						0.71
9	Blanco Chico	847.40	711.10						0.86
10 *	Napo	562.30	134.07	0.65	0.36	0.66	238	4.69	0.77
11 *	Jivino Azul	91.00	202.45	0.52	0.39	0.93	216	4.57	0.55
12	Pucacachi	26.23	44.95	1.08	0.27	0.09			0.00
13 *	Jandiyacu	195.73	223.70	0.46	0.41	1.22	281	4.87	0.00
14	Conambo	24.40	17.55						0.28
15	Harbent	74.33	22.35						0.26
16	La Sur	144.87	55.65	0.32	0.59	1.97			0.37
17	Manduro	70.20	62.65						0.28
18	Pisuri	183.87	90.47	0.49	0.17	0.55			0.38
19 *	Sacha	44.63	41.30	0.27	0.59	1.53	246	4.60	0.34
20	Huamayacu	100.60	35.80						0.70
21	Blanco	62.13	30.90						0.43
22	Huachito	32.70	119.10						0.43
23 *	Indillana	431.13	469.80	0.62	1.34	1.76	243	4.96	0.67
24	Blanco Grande	34.60	97.70	1.35	0.61	0.67			0.57
Well Aguarico 4	Tena province			0.08	0.01	0.18			

## Data Availability

Raw data of the sequence reads are available at the official website of the National Center for Biotechnology Information using the following url: http://www.ncbi.nlm.nih.gov/bioproject/1054535 (accessed on 19 December 2023).

## Supplementary Materials

The following supporting information can be downloaded at: https://www.mdpi.com/article/10.3390/toxics12020119/s1, Figure S1: Microbial diversity (ASVs) at a family level in each sediment sample (left axis) and the Polycyclic Aromatic Hydrocarbon concentrations (PAH, mg·kg^−1^) in each sample (right axis).
