# Supplementary material for: Bacterial Diversity in Old Hydrocarbon Polluted Sediments of Ecuadorian Amazon River Basins
Source: Toxics. 2024 Jan 31;12(2):119. doi: 10.3390/toxics12020119 (PMC10892221; doi:10.3390/toxics12020119)
Supplement: Supplementary file 1 [file toxics-12-00119-s001.zip › toxics-2812616-supplementary.pdf]

Suplementary Materials

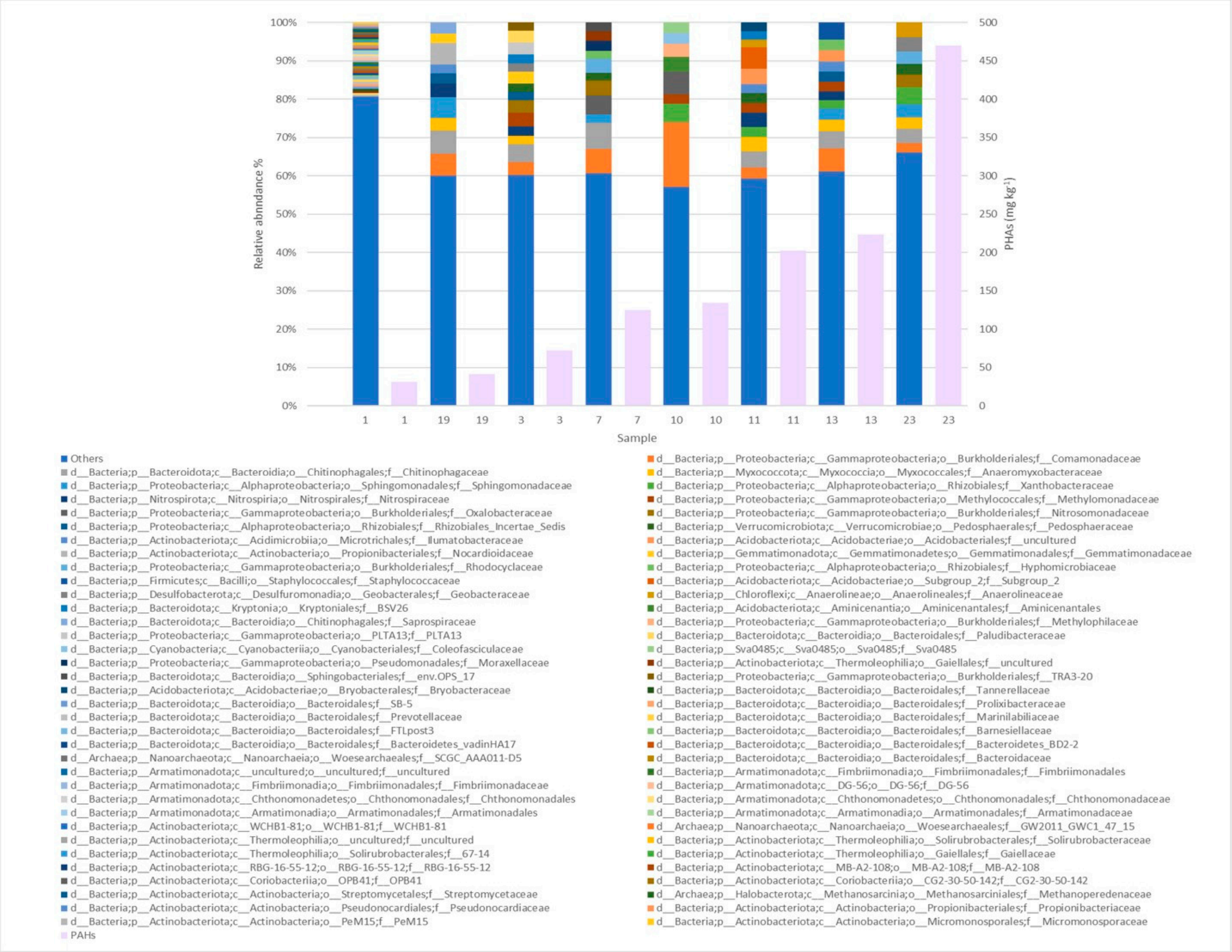

Figure S1: Microbial diversity (ASVs) at a family level in each sediment sample (left axis) and the Polycyclic Aromatic Hydrocarbon concentrations (PAH, mg·kg^{-1}) in each sample (right axis).
